# Changes in Fruit Characteristics of Various Blueberry Cultivars During Ripening Stages

**DOI:** 10.3390/foods15122157

**Published:** 2026-06-15

**Authors:** Jing Xiong, Yufang Xu, Shiyu He, Xiya Hong, Bingying Zhang, Xinli Ouyang, Qiyuan Zhang, Fu Wan

**Affiliations:** 1 School of Forestry, Central South University of Forestry and Technology, Changsha 410004, China; 20230288@csuft.edu.cn (J.X.); 18774998579@163.com (S.H.); 20230290@csuft.edu.cn (Q.Z.); 2 Hunan Testing Institute of Product and Commodity, Changsha 410007, China; hnqixuyuf@126.com; 3 School of Food Science and Engineering, Central South University of Forestry and Technology, Changsha 410004, China; 18075180797@163.com (X.H.); 17736314301@163.com (B.Z.); 15932866225@163.com (X.O.)

**Keywords:** blueberry fruit, ripening progress, fruit quality, organic acid, soluble sugar

## Abstract

The quality of fresh blueberries is determined by physicochemical properties and sugar–acid composition, which exhibit significant variations across different cultivars and ripening stages. This study investigated two rabbiteye blueberry cultivars (‘Brightwell’, ‘Homebell’) and three highbush blueberry cultivars (‘Bluegold’, ‘Emerald’, ‘Legacy’), measuring basic physicochemical indices, soluble sugar and organic acid profiles at six ripening stages. HPLC analysis revealed that soluble sugars in blueberries are primarily composed of fructose and glucose. The organic acids in highbush blueberries are dominated by malic and citric acids, while rabbiteye blueberries contain higher levels of quinic acid in addition to malic and citric acids. Correlation analysis demonstrated that glucose and fructose contents were significantly positively correlated (*p* ≤ 0.01) with average fruit weight, soluble pectin to total pectin ratio (SP/TP), and total soluble solids to titratable acidity ratio (TSS/TA), but showed a highly significant negative correlation with malic acid. In contrast, malic acid exhibited a significant positive correlation (*p* ≤ 0.05) with firmness and moisture content. Cluster analysis divided the measured parameters into two groups: Class 1 includes average fruit weight, SP/TP, TSS/TA, and soluble sugars. Class 2 comprises fruit shape index, firmness, moisture content, and organic acids. Quality variation patterns among blueberry cultivars clustered according to cultivated type (rabbiteye and highbush), with differences within the same type being smaller than those between the two types. Rabbiteye blueberries exhibited a higher TSS/TA and a sweeter taste, whereas highbush varieties displayed softer texture, higher moisture content, and a balanced sweet–sour juiciness. This study clarified the dynamics of sugar and acid metabolism during blueberry ripening, providing a theoretical basis for quality evaluation, harvest timing, and breeding.

## 1. Introduction

Blueberry (*Vaccinium* spp.) is a deciduous shrub belonging to the Ericaceae family. Its fruit is known for a pleasant sweet and sour taste and is rich in various vitamins, minerals, dietary fiber, anthocyanins and other bioactive components [1], which contribute to multiple health benefits, including antioxidant, anti-inflammatory, anticancer, cardiovascular disease prevention and neuroprotective effects [2,3,4]. Consequently, blueberries have been recognized by the Food and Agriculture Organization as one of the “five major healthy foods for humans” [5]. The main types of blueberries available on the market include highbush blueberry (*V*. *corymbosum* L.), rabbiteye blueberry (*V*. *ashei*), and lowbush blueberry (*V. angustifolium*), although many modern cultivars are interspecific hybrids [6]. Highbush blueberry is the most widely cultivated and commercially significant type globally, characterized by a balanced sweet and sour taste and dominated the fresh blueberry market. The rabbiteye blueberry group includes most of the cultivars planted in subtropical regions, known for its sweet taste and good storability [7]. Highbush cultivars such as ‘Bluecrop’ generally produce medium to large fruit with a balanced sweet–sour taste and softer texture, whereas rabbiteye cultivars like ‘Tifblue’ typically have smaller to medium fruit, higher firmness, higher soluble solids content and sugar/acid ratio, which contribute to better postharvest storage and a sweeter flavor profile [8]. With the continuous expansion of the blueberry consumption market and the parallel increase in breeding programs, consumers are exposed to an increasing variety of blueberry cultivars, leading to progressively higher expectations for their fresh-eating flavor.

The content, type, and proportion of sugars and organic acids are the key factors determining the taste of blueberries. The sweetness of fruit is primarily determined by soluble sugar content, among which fructose has the highest relative sweetness value, being 2.3 times sweeter than glucose and 5.2 times sweeter than sucrose [8,9]. Zheng et al. [10] reported that glucose accounts for 51.13~62.94% of total sugars in blueberry fruit, fructose for 36.21~45.40%, and sucrose for only 0.43~4.42%. The content of organic acids in fruit affects its taste and flavor. In berries, the main organic acids include citric, malic, and quinic acids, with citric acid comprising 30~95% of total organic acids [11,12]. However, different organic acids differ in sourness intensity. Mao et al. [13] used citric acid as a reference to determine the relative sourness indices of other organic acids, reporting that malic acid has an index of 0.74, indicating the highest sourness intensity. The sugar/acid ratio is an important factor determining blueberry taste. Previous studies have reported that ‘Elliott’ and ‘Bluegold’ blueberries exhibit a pronounced sour taste and are high-acid retaining varieties, while ‘Jersey’ and ‘Earliblue’ blueberries have a higher sugar/acid ratio and are distinctly sweeter [10]. Besides influencing taste, the sugar/acid ratio also significantly affects the basic physicochemical quality of fruit. Yin et al. [14] found that the sugar/acid ratio in pear fruit is significantly positively correlated with single fruit weight, and glucose content is positively correlated with fruit crispness. Similarly, during apple fruit development, the decrease in titratable acidity parallels the decline in fruit firmness [15]. However, the correlation between sugar/organic acid profiles and basic physicochemical indicators in blueberry fruit has not yet been reported.

During fruit ripening, the basic physicochemical indices and sugar–acid components of fruit undergo substantial changes, and studying these changes provides a theoretical basis for evaluating fruit maturity and optimizing taste [16]. Yang et al. [17] measured the physiological characteristics, soluble sugars, and organic acids of apples at different ripening stages and found that from 60 to 180 days after full bloom, fruit firmness decreased significantly (from 19.45 to 6.27 kg/cm^2^), the contents of fructose and sucrose increased (from 28.75 to 55.24 mg/g FW and from 4.70 to 32.07 mg/g FW, respectively), and malic acid content decreased markedly (from 6.90 to 2.81 mg/g FW). Mo et al. [18] determined fruit quality parameters of wampee at five ripening stages and found that from stage 1 to stage 5, total organic acid content decreased (from 11.74 to 7.40 mg/g), with citric acid decreasing from 7.97 to 5.83 mg/g; total soluble sugar content increased significantly (from 24.64 to 76.02 mg/g), with sucrose increasing from 11.55 to 40.00 mg/g. Similar to apples and wampee, blueberry fruits undergo dynamic changes in physiological and biochemical characteristics, especially in the composition and content of soluble sugars and organic acids, with skin color turning from green to blue during cell wall disassembly [1]. However, most current research has focused on the physicochemical quality, antioxidant activity, and postharvest storage conditions of mature blueberries, neglecting the dynamic changes in basic physicochemical indices and sugar–acid components during growth, as well as interspecific differences. Zhang et al. [10] detected the sugar–acid composition of ripe fruit from 11 highbush blueberry cultivars and found significant differences in sugar and acid contents between different production regions. Zhang et al. [19] reported that the main antioxidant compounds in ripe highbush blueberries are chlorogenic acid, ascorbic acid, citric acid, and catechin, and explored the correlation between soil pH and the contents of anthocyanins and organic acids. Zhang et al. [20] analyzed the accumulation patterns of flavonoids, anthocyanins, and phenolic acids in ‘Legacy’ blueberries at three developmental stages. Dragišić Maksimović et al. [21] investigated changes in fruit quality of highbush blueberries under different storage conditions and found that cultivar was a primary factor influencing fruit texture, sugar–acid content, and phenolic compounds, providing a basis for selecting blueberry varieties with superior quality under specific storage conditions. Therefore, studying the physiological and biochemical characteristics of blueberry fruit during ripening, especially the composition and content of soluble sugars and organic acids, is of great significance for evaluating fruit quality, determining harvest timing, and guiding cultivation and breeding.

This study determined the basic indicators of blueberry fruits at different growth stages, as well as the dynamic changes in the content and composition of sugars and organic acids. It also compared the differences in the content and composition of organic acids and sugars among different blueberry varieties. At the same time, the influence of blueberry varieties and growth stages on quality indicators was evaluated through analysis of variance (ANOVA), correlation analysis, and cluster analysis. The aim of this study is to provide theoretical and practical foundations for the quality evaluation, optimization, and breeding of fresh blueberries.

## 2. Materials and Methods

### 2.1. Plant Material

**Figure 1:**
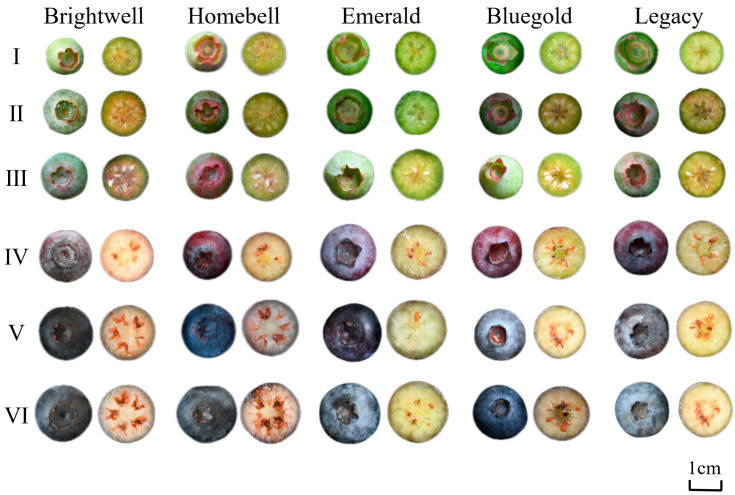
Fruits of ‘Brightwell’, ‘Homebell’, ‘Emerald’, ‘Bluegold’, and ‘Legacy’ blueberry cultivars in six ripening stages (I: green fruit at early stage; II: green fruit at expanding stage; III: large green fruit; IV: pink fruit at early ripening; V: purple fruit at intermediate ripening; VI: dark purple fruit at full ripening).

Five blueberry cultivars were selected for this study: two rabbiteye varieties (‘Brightwell’ and ‘Homebell’) and three highbush varieties, including the northern highbush ‘Bluegold’ and the southern highbush ‘Emerald’ and ‘Legacy’. The experiment adopted a completely randomized design. Five 12-year-old plants with the same structure and management level were selected for each variety for the collection of blueberry fruit samples. The fruits were harvested from the Ningxiang Blueberry Base in Changsha City, Hunan Province from May to July in 2025. Blueberry fruits from six developmental stages were collected from marked trees: Stage I (green skin, early fruiting stage, collected 23~37 days after full bloom), Stage II (green skin, early fruiting stage, collected 33~47 days after full bloom), Stage III (green fruit, large fruiting stage, collected 43~57 days after full bloom), Stage IV (pink skin, slightly ripe stage, collected 55~67 days after full bloom), Stage V (slightly purple skin, moderately ripe stage, collected 58~69 days after full bloom), Stage VI (deep purple skin, fully ripe stage, collected 60~75 days after full bloom). The detailed harvest timing for each cultivar is provided in Supplementary Table S1. Blueberry fruits with similar maturity conditions, size, and free from pests and diseases were randomly selected. During sampling, the integrity of the external fruit powder on the fruit skin was preserved as much as possible. The sampling amount for each variety and each maturity stage was approximately 100 g. After sample collection, the samples were divided into two portions. One portion was immediately used for basic index determination, and the other was quickly frozen with liquid nitrogen at −80 °C and stored in a refrigerator for 100 days to be used for the determination of sugars and organic acids. The size, shape, and color of blueberries of different varieties at different growth stages are shown in Figure 1.

### 2.2. Reagents

Galacturonic acid, glucose, fructose, sucrose, malic acid, citric acid, shikimic acid, quinic acid, and oxalic acid standards were purchased from Sigma-Aldrich (St. Louis, MO, USA). Other reagents were of analytical grade and provided by Sinopharm Chemical Reagent Co., Ltd. (Shanghai, China).

### 2.3. Measurement of Blueberry Physicochemical Quality

For each cultivar and ripening stage, 30 blueberry fruits were randomly selected, and the fruit weight was measured using an electronic balance (ME204E, Mettler Toledo, Shanghai, China) and the average value was calculated.

Fruit shape index was calculated as the ratio of longitudinal diameter to transverse diameter. The longitudinal and transverse diameters of 30 randomly selected fruits per replicate were measured using a digital vernier caliper (Mitutoyo Corp., Kawasaki, Japan).

Fruit firmness was measured directly using a portable fruit hardness tester equipped with a cylindrical probe (3.5 mm diameter) (GY-2, Wenzhou Wei Electronics Co., Ltd., Wenzhou, China). The probe was inserted perpendicularly into the equatorial region of the fruit, and the peak force required to penetrate the fruit skin was recorded in N.

The contents of TSS and TA were measured using a fruit sugar–acid meter (PAL-BX/ACID F5, Atago Scientific Instruments Co., Ltd., Guangzhou, China), and the results were expressed as %.

The moisture content was determined using the direct drying method in accordance with GB 5009.3-2016 [22]: samples were dried in an electric thermostatic drying oven (DHG-9070A, Shanghai Yiheng Scientific Instrument Co., Ltd., Shanghai, China) at 101~105 °C to constant weight, and the moisture content was calculated from the mass loss and the results were expressed as a percentage. Detailed measurement procedures are provided in Supplementary Material Note S1.

### 2.4. Determination of Pectin Content

Soluble pectin (SP) and total pectin (TP) were determined by the carbazole colorimetric method following the industry standard NY/T 2016-2011 [23] and Chea et al. [24], with galacturonic acid as the standard. In brief, 2.5 g of blueberry homogenate was mixed with 35 mL of 75 °C anhydrous ethanol, heated using a constant temperature water bath (SHHW 21-420, Beijing Ever Bright Medical Treatment Instrument Co., Ltd., Beijing, China) at 85 °C for 10 min, and centrifuged at 4000 rpm for 15 min using a centrifuge (H1650, Hunan Cence Instrument Co., Ltd., Changsha, China). The precipitate was washed repeatedly with 67% ethanol until free of sugar (negative α-naphthol test). For TP extraction, the precipitate was hydrolyzed with pH 0.5 sulfuric acid at 85 °C for 60 min, then diluted to 100 mL. For SP extraction, the precipitate was extracted with hot water at room temperature for 30 min, then diluted to 100 mL. An aliquot (1.0 mL) of the filtrate or galacturonic acid standard solution (0~100 mg/L) was mixed with 0.25 mL of 1 g/L carbazole in ethanol, followed by 5.0 mL concentrated sulfuric acid. After reaction at 85 °C for 20 min and cooling, absorbance was measured at 525 nm using a UV-Vis spectrophotometer (UV1200, Shanghai Mapada Instruments Co., Ltd., Shanghai, China). Pectin content was calculated from a standard curve and expressed as mg galacturonic acid equivalent per 100 g fresh weight (mg GAE/100 g FW). Detailed procedures are given in Supplementary Material Note S2.

### 2.5. Determination of Sugars and Organic Acids in Blueberries

#### 2.5.1. Determination of Sugars

Sugars were extracted using the method described by Li et al. [25], with some modifications. A total of 0.5 g of blueberry homogenate was mixed with 9 mL of 60% (*v*/*v*) ethanol and extracted by an ultrasonic cleaner (SB-5200DTD, Ningbo Scientz Biotechnology Co., Ltd., Ningbo, China) at 25 °C and 360 W for 30 min. After centrifugation at 11,000 rpm for 15 min, the supernatant was collected, and the extraction was repeated twice. The pooled extracts were diluted to 25 mL with deionized water, filtered through a 0.22 μm syringe filter, and placed into a 2 mL vial for HPLC analysis (Agilent 6460, Agilent Technologies, Santa Clara, CA, USA).

Fructose, sucrose, and glucose were selected as soluble sugar standards. Each sugar (5.00 mg) was accurately weighed and dissolved in 10 mL of deionized water. Appropriate volumes of each sugar solution were mixed and diluted to prepare mixed standard solutions at concentrations of 20, 50, 80, 100, 150, 200, and 250 μg/mL for each sugar. Sugars were identified by retention times and quantified by peak areas using the corresponding standard curves. Linear regression equations and correlation coefficients were calculated by plotting the peak areas against the corresponding concentrations of the standards.

The chromatographic conditions for sugar analysis were optimized based on the method reported by Zhang et al. [19] with some modifications. The mobile phase consisted of acetonitrile and water at a volume ratio of 70:30, and separation was achieved using a XBridge BEH Amide column (5 μm, 4.6 × 250 mm, Waters, Milford, MA, USA). The column temperature was maintained at 40 °C, the injection volume was 20 μL, and the flow rate was 1.0 mL/min. An evaporative light scattering detector (Agilent 1290 Infinity, Agilent Technologies, Santa Clara, CA, USA) was operated under the following conditions: drift tube temperature 80~90 °C, nitrogen pressure 350 kPa, and impactor off. Chromatograms were recorded over 30 min.

#### 2.5.2. Determination of Organic Acids

Organic acids were extracted according to the method described by Li et al. [25] with slight modifications. Blueberry fruits were homogenized in a mortar, and 0.5 g of the homogenate was mixed with 9 mL of deionized water. The mixture was ultrasonically extracted at 25 °C and 360 W for 30 min, followed by centrifugation at 11,000 rpm for 15 min. The supernatant was collected, and the extraction process was repeated twice. The combined supernatants were adjusted to a final volume of 20 mL with deionized water, filtered through a 0.22 μm syringe filter, and transferred into a 2 mL vial for HPLC analysis.

Oxalic acid, citric acid, malic acid, shikimic acid, and quinic acid were selected as organic acid standards. Each standard (5.00 mg) was accurately weighed and dissolved in 1 mL of deionized water. Appropriate volumes of each organic acid solution were mixed and diluted to prepare mixed standard solutions at the following concentrations: oxalic acid and shikimic acid at 1.00, 2.00, 5.00, 8.00, 10.00, and 20.00 μg/mL; citric acid, malic acid, and quinic acid at 50, 80, 100, 200, 500, and 1000 μg/mL.

For the determination of oxalic acid and shikimic acid, the procedure described by Zhang et al. [19] was used with some modifications. The mobile phase comprised solvent A (0.01 mol/L potassium dihydrogen phosphate adjusted to pH 2.6 with phosphoric acid) and solvent B (methanol). Isocratic elution was performed with 97% A and 3% B at a flow rate of 0.8 mL/min. The column temperature was set at 30 °C, the injection volume was 10 μL, and detection was carried out at 214 nm. For the determination of citric acid, malic acid, and quinic acid were analyzed according to the method of Tao et al. [15] with some modifications. The mobile phase consisted of 0.1% phosphoric acid (solvent A) and methanol (solvent B). Isocratic elution was performed with 75% A and 25% B at a flow rate of 0.8 mL/min. The column temperature was maintained at 40 °C, the injection volume was 20 μL, and the multi-wavelength detector (Agilent 1260 Infinity III, Agilent Technologies, USA) wavelength was set at 210 nm.

### 2.6. Statistical Analysis

For each cultivar and ripening stage, three samples were prepared by pooling fruits from the five plants. All measurements were performed once per sample, resulting in three replicates (*n* = 3) for each parameter. Results are expressed as mean ± standard deviation (SD) of the triplicates. Data were processed using Excel 2019. Analysis of variance (ANOVA) was performed with SPSS 27.0, and significant differences among means were determined using Duncan’s multiple range test at a 95% confidence level. All figures were created using Origin 2024.

## 3. Results

### 3.1. Physicochemical Qualities of Five Blueberries Under Different Growth Stages

#### 3.1.1. Average Fruit Weight, Fruit Shape Index and Moisture Content of Five Blueberry Cultivars

Table 1 presents the changes in average fruit weight, fruit shape index, and moisture content of different blueberry cultivars at various growth stages. As shown in Table 1, significant differences (*p* < 0.05) in average fruit weight were observed among cultivars at each developmental stage. Notably, average fruit weight increased rapidly as the fruit developed. Among the cultivars, ‘Emerald’ exhibited the greatest increase, with its average fruit weight significantly surpassing the other cultivars from stage II. The average fruit weight of its mature fruits was 96.08%, 109.79%, 114.29%, and 35.14% higher than that of the other cultivars respectively. Throughout the growing process, average fruit weight increased progressively at each stage without stagnation.

The fruit shape index, the ratio of longitudinal diameter to transverse diameter, reflects fruit roundness, with a higher value indicating a rounder shape. According to Table 1, the fruit shape index of the five cultivars generally showed a decreasing trend, indicating that blueberry fruits gradually transition from round to flat shapes during maturation. ‘Bluegold’ consistently maintained the highest values (0.95~0.91), which were significantly higher than those of the other cultivars (*p* < 0.05). It was 16.67%, 18.18%, 31.88%, and 26.39% higher than that of the other four cultivars, suggesting that this cultivar produces rounder fruits. In contrast, ‘Emerald’ had the lowest values across all growing stages (0.78~0.69), reflecting its flatter fruit shape.

As shown in Table 1, the moisture content of mature blueberry fruits ranged from 78.38% to 85.95%. Among the cultivars, ‘Brightwell’ had the lowest moisture content, indicating the highest dry matter accumulation, followed by ‘Homebell’. Both belong to the rabbiteye blueberry type, which matures later than highbush cultivars and therefore accumulates more dry matter in the fruit. In contrast, highbush blueberries exhibited higher moisture content than rabbiteye blueberries.

**Table 1:** Single fruit weight, fruit shape index and moisture content of five blueberry cultivars across six ripening stages.

Parameters	Cultivars	Ripening Stage					
		I	II	III	IV	V	VI
Average fruit weight (g)	Brightwell	0.22 ± 0.01 ^Fd^	0.59 ± 0.05 ^Eb^	0.72 ± 0.01 ^Db^	1.24 ± 0.03 ^Cc^	1.36 ± 0.02 ^Bc^	1.53 ± 0.02 ^Ac^
	Homebell	0.26 ± 0.00 ^Fc^	0.38 ± 0.02 ^Ec^	0.68 ± 0.02 ^Dc^	0.81 ± 0.02 ^Cd^	0.89 ± 0.03 ^Be^	1.43 ± 0.02 ^Ad^
	Emerald	0.20 ± 0.01 ^Fe^	0.74 ± 0.01 ^Ea^	1.05 ± 0.03 ^Da^	2.42 ± 0.06 ^Ca^	2.50 ± 0.05 ^Ba^	3.00 ± 0.10 ^Aa^
	Bluegold	0.27 ± 0.01 ^Fb^	0.41 ± 0.01 ^Ec^	0.58 ± 0.01 ^Dd^	0.81 ± 0.01 ^Cd^	0.96 ± 0.03 ^Bd^	1.40 ± 0.03 ^Ad^
	Legacy	0.32 ± 0.00 ^Fa^	0.56 ± 0.01 ^Eb^	0.72 ± 0.01 ^Db^	1.43 ± 0.02 ^Cb^	1.47 ± 0.02 ^Bb^	2.22 ± 0.03 ^Ab^
Fruit shape index	Brightwell	0.82 ± 0.04 ^Ab^	0.79 ± 0.03 ^Bc^	0.79 ± 0.03 ^Bbc^	0.78 ± 0.03 ^Bbc^	0.76 ± 0.03 ^Bc^	0.78 ± 0.04 ^Bb^
	Homebell	0.93 ± 0.05 ^Aa^	0.89 ± 0.04 ^Ab^	0.80 ± 0.05 ^Bb^	0.81 ± 0.01 ^Bb^	0.80 ± 0.02 ^BCb^	0.77 ± 0.04 ^Cb^
	Emerald	0.78 ± 0.04 ^Ac^	0.72 ± 0.03 ^Bd^	0.69 ± 0.05 ^Be^	0.70 ± 0.03 ^Be^	0.70 ± 0.03 ^Bd^	0.69 ± 0.01 ^Bd^
	Bluegold	0.95 ± 0.03 ^Aa^	0.94 ± 0.02 ^Aa^	0.90 ± 0.04 ^Ba^	0.89 ± 0.03 ^Ba^	0.88 ± 0.03 ^Ba^	0.91 ± 0.04 ^Ba^
	Legacy	0.78 ± 0.02 ^ABc^	0.79 ± 0.04 ^Ac^	0.75 ± 0.03 ^Cd^	0.77 ± 0.02 ^BCcd^	0.75 ± 0.02 ^Cc^	0.72 ± 0.02 ^Dc^
Moisture content (%)	Brightwell	89.51 ± 0.08 ^Ac^	87.98 ± 0.12 ^Bd^	82.60 ± 0.43 ^Ce^	82.46 ± 0.44 ^Cc^	80.78 ± 0.41 ^Dd^	78.38 ± 0.69 ^Ee^
	Homebell	88.65 ± 0.05 ^Ae^	86.37 ± 0.18 ^Be^	84.56 ± 0.22 ^Cd^	84.38 ± 0.11 ^Cb^	83.57 ± 0.36 ^Dc^	81.39 ± 0.33 ^Ed^
	Emerald	90.07 ± 0.15 ^Ba^	91.09 ± 0.08 ^Aa^	87.41 ± 0.10 ^Da^	87.89 ± 0.11 ^Ca^	86.18 ± 0.16 ^Ea^	85.95 ± 0.08 ^Fa^
	Bluegold	89.88 ± 0.07 ^Ab^	89.74 ± 0.04 ^Ab^	86.15 ± 0.50 ^Bc^	84.00 ± 0.15 ^Cb^	83.61 ± 0.22 ^Cc^	82.83 ± 0.25 ^Dc^
	Legacy	89.34 ± 0.02 ^Ad^	89.36 ± 0.11 ^Ac^	86.66 ± 0.16 ^Cb^	87.75 ± 0.33 ^Ba^	85.05 ± 0.68 ^Db^	84.42 ± 0.36 ^Eb^

Results are expressed as mean ± SD. Different lowercase letters indicate significant differences in the same growing stage among different cultivars, while different capital letters indicate that there are significant differences in the same cultivar among the different growing stages (*p* < 0.05).

#### 3.1.2. The Fruit Firmness, Total Pectin Content and Soluble Pectin Content of Five Blueberry Cultivars

The changes in fruit firmness and pectin content of blueberries across different growth stages are given in Table 2. As the fruit matured, firmness initially increased and then decreased sharply at the early coloring stage (Stage IV). Among all mature cultivars, the firmness of ‘Brightwell’, ‘Homebell’, ‘Emerald’ and ‘Legacy’ exceeded 7 N, with no significant differences (*p* > 0.05), while ‘Bluegold’ exhibited the lowest firmness at 5.86 N.

As the fruit ripened, the soluble pectin (SP) content gradually increases, with the largest increment observed during the coloring stage (Stages IV~VI). At full maturity, the SP contents of the five cultivars were 7.72, 23.07, 3.31, 18.67, and 5.63 times higher than those at Stage I, respectively, and the SP content of rabbiteye blueberries was generally higher than that of highbush cultivars (‘Emerald’, ‘Bluegold’, and ‘Legacy’). The total pectin (TP) content differed significantly between rabbiteye and highbush blueberries (*p* < 0.05). During early growth (Stages I~II), the TP content of rabbiteye blueberries decreased, then increased rapidly, reaching levels at maturity that were 46.35% and 75.39% higher than those at Stage II, respectively. In contrast, the TP content of highbush blueberries showed an overall declining trend, decreasing by 39.3%, 15.5%, and 36.3% at maturity compared to Stage I, respectively.

The SP/TP ratio is a key indicator of fruit maturity and textural softening. As maturity progressed, the SP/TP ratio generally increased in all cultivars, with ‘Bluegold’ showing the greatest increase of 34.34%. Due to the increase in TP content during the late stage in the rabbiteye blueberries, their SP/TP ratios were slightly lower than those of the highbush cultivars, indicating that rabbiteye blueberries have a firmer texture than highbush blueberries, which is consistent with the firmness measurements.

**Table 2:** The fruit firmness, total pectin content and soluble pectin content of five blueberry cultivars across six ripening stages.

Parameters	Cultivars	Growing Stages					
		I	II	III	IV	V	VI
Firmness (N)	Brightwell	24.74 ± 2.29 ^Cb^	39.08 ± 3.70 ^Bab^	49.52 ± 4.99 ^Aa^	11.26 ± 1.43 ^Dab^	10.02 ± 0.88 ^Dab^	7.52 ± 0.82 ^Da^
	Homebell	24.96 ± 2.10 ^Bb^	35.12 ± 4.17 ^Abc^	33.38 ± 2.16 ^Acd^	12.18 ± 1.65 ^Cab^	11.34 ± 1.29 ^Ca^	7.28 ± 0.62 ^Da^
	Emerald	21.16 ± 1.97 ^Cc^	33.68 ± 3.41 ^Bcd^	42.42 ± 7.44 ^Ab^	12.92 ± 2.02 ^Da^	9.10 ± 0.16 ^DEb^	7.08 ± 0.96 ^Ea^
	Bluegold	24.14 ± 1.84 ^Bbc^	21.06 ± 2.02 ^Be^	33.9 ± 5.63 ^Ad^	11.14 ± 2.37 ^Cab^	7.66 ± 1.07 ^CDc^	5.86 ± 0.11 ^Db^
	Legacy	48.64 ± 3.46 ^Aa^	41.18 ± 4.05 ^Ba^	43.18 ± 1.34 ^Bb^	9.56 ± 1.67 ^Cb^	9.04 ± 1.75 ^Cb^	7.76 ± 0.59 ^Ca^
Soluble pectin content (mg (GAE)/100 g FW)	Brightwell	32.55 ± 1.93 ^Fc^	51.84 ± 6.50 ^Eb^	118.31 ± 2.45 ^Da^	138.66 ± 2.77 ^Ca^	203.40 ± 4.03 ^Ba^	251.41 ± 3.74 ^Aa^
	Homebell	10.70 ± 2.83 ^Fc^	22.11 ± 2.45 ^Ed^	93.95 ± 2.83 ^Db^	138.04 ± 2.33 ^Ca^	168.26 ± 6.47 ^Bb^	246.88 ± 4.24 ^Ab^
	Emerald	59.11 ± 6.98 ^Ca^	64.62 ± 6.50 ^Da^	77.65 ± 4.75 ^Cc^	82.43 ± 1.85 ^Cc^	116.67 ± 1.41 ^Be^	195.91 ± 1.41 ^Ad^
	Bluegold	11.62 ± 4.66 ^Ec^	15.94 ± 2.97 ^Ee^	72.98 ± 1.85 ^Dd^	85.62 ± 3.50 ^Cb^	137.42 ± 3.74 ^Bc^	216.97 ± 2.83 ^Ac^
	Legacy	34.13 ± 3.70 ^Db^	36.29 ± 2.33 ^Dc^	34.13 ± 2.45 ^De^	69.28 ± 1.85 ^Cd^	120.16 ± 1.85 ^Bd^	192.29 ± 1.41 ^Ae^
Total pectin content (mg (GAE)/100 g FW)	Brightwell	640.11 ± 11.10 ^Ee^	550.59 ± 4.66 ^Ed^	729.94 ± 8.34 ^Cb^	637.44 ± 7.70 ^Db^	827.98 ± 11.30 ^Aa^	805.78 ± 8.74 ^Bb^
	Homebell	703.42 ± 5.65 ^Cc^	475.26 ± 7.40 ^Ee^	755.22 ± 4.66 ^Ba^	655.32 ± 8.34 ^Da^	760.77 ± 8.34 ^Bb^	833.54 ± 8.74 ^Aa^
	Emerald	792.83 ± 4.66 ^Aa^	684.94 ± 6.50 ^Ba^	662.85 ± 8.48 ^Bc^	581.89 ± 5.65 ^Cc^	558.32 ± 4.89 ^Dc^	569.32 ± 3.85 ^Ed^
	Bluegold	695.40 ± 8.48 ^Ad^	575.77 ± 6.50 ^Cc^	599.20 ± 8.48 ^Bd^	506.71 ± 10.30 ^Ed^	549.87 ± 3.85 ^Dd^	602.29 ± 5.65 ^Bc^
	Legacy	748.44 ± 5.95 ^Ab^	602.29 ± 4.66 ^Bb^	477.72 ± 5.65 ^De^	475.87 ± 3.85 ^De^	431.47 ± 2.83 ^Ee^	549.26 ± 4.89 ^Ce^
Soluble pectin/Total pectin (%)	Brightwell	5.09 ± 0.50 ^Fb^	9.30 ± 1.54 ^Eb^	16.21 ± 0.42 ^Da^	21.76 ± 0.66 ^Ca^	24.57 ± 0.57 ^Bb^	34.93 ± 0.62 ^Ac^
	Homebell	1.52 ± 0.39 ^Fd^	4.65 ± 0.57 ^Ed^	12.44 ± 0.30 ^Db^	21.06 ± 0.55 ^Ca^	22.12 ± 0.74 ^Bc^	29.62 ± 0.28 ^Ad^
	Emerald	7.47 ± 0.85 ^Ea^	9.43 ± 0.86 ^Fa^	11.71 ± 0.56 ^Dc^	14.17 ± 0.28 ^Cd^	20.88 ± 0.41 ^Bd^	34.41 ± 0.52 ^Ac^
	Bluegold	1.68 ± 0.69 ^Fd^	2.77 ± 0.54 ^Ee^	12.18 ± 0.44 ^Db^	16.90 ± 0.51 ^Cb^	24.99 ± 0.63 ^Bb^	36.02 ± 0.29 ^Aa^
	Legacy	4.56 ± 0.53 ^Fc^	6.03 ± 0.43 ^Ec^	7.14 ± 0.44 ^Dd^	14.56 ± 0.28 ^Cc^	27.85 ± 0.25 ^Ba^	35.02 ± 0.50 ^Ab^

Results are expressed as mean ± SD. Different lowercase letters indicate significant differences in the same growing stage among different cultivars, while different capital letters indicate that there are significant differences among the same cultivar in the different growing stages (*p* < 0.05).

#### 3.1.3. TA, TSS and TSS/TA of Five Blueberry Cultivars

Table 3 presents the changes in taste-related indicators of the five blueberry cultivars during fruit development. Throughout fruit ripening, total soluble solids (TSSs) content showed an overall increasing trend, with two notable increases occurring at the onset of fruit coloration (Stage IV) and near full maturity (Stage VI). At full maturity, the TSS contents of ‘Brightwell’ and ‘Homebell’ were 19.10% and 15.00%, respectively, which were significantly higher than those of the highbush cultivars (‘Bluegold’: 12.04%; ‘Emerald’: 10.78%; ‘Legacy’: 11.22%). Titratable acidity (TA) first increased and then decreased, peaking at the large green fruit stage (Stage III) and reaching its minimum at full maturity (Stage VI). Among the five ripe blueberry cultivars, ‘Bluegold’ exhibited the lowest value in TA (0.594%), and significant differences among cultivars were observed at the same developmental stage.

The TSS/TA ratio directly reflects the sweetness of blueberry fruit, with a higher value indicating a sweeter taste. As shown in Table 3, during Stages I~III, due to the increase in TA content, the TSS/TA ratios of the five cultivars generally decreased, with reductions of 18.99%, 10.03%, 72.60%, 30.25%, and 50.70%, respectively. Subsequently, during the coloring stages (Stages IV~VI), the TSS/TA ratios gradually increased, reaching levels at maturity that were 11.62, 7.24, 4.59, 8.57, and 6.32 times those at Stage I, respectively. Among the cultivars, ‘Brightwell’ exhibited the highest TSS/TA ratio (29.40%), significantly higher than the other cultivars, indicating its superior sweetness.

**Table 3:** TA, TSS and TSS/TA of five blueberry cultivars across six ripening stages.

Parameters	Cultivars	Growing Stages					
		I	II	III	IV	V	VI
TSS (%)	Brightwell	5.16 ± 0.11 ^Eb^	4.78 ± 0.26 ^Ec^	6.04 ± 0.18 ^Db^	13.16 ± 0.29 ^Ca^	13.64 ± 0.27 ^Ba^	19.10 ± 0.54 ^Aa^
	Homebell	5.94 ± 0.21 ^Da^	6.48 ± 0.19 ^Ca^	6.42 ± 0.15 ^Ca^	11.10 ± 0.32 ^Bb^	11.40 ± 0.26 ^Bb^	15.00 ± 0.32 ^Ab^
	Emerald	4.22 ± 0.26 ^Dc^	4.44 ± 0.27 ^Dc^	5.34 ± 0.17 ^Cc^	9.36 ± 0.23 ^Bd^	9.46 ± 0.21 ^Bde^	10.78 ± 0.32 ^Ad^
	Bluegold	5.40 ± 0.22 ^Db^	5.44 ± 0.11 ^Db^	6.08 ± 0.19 ^Cb^	10.18 ± 0.69 ^Bc^	10.60 ± 0.52 ^Bc^	12.04 ± 0.23 ^Ac^
	Legacy	5.26 ± 0.09 ^Db^	5.08 ± 0.41 ^Dc^	4.64 ± 0.18 ^Ed^	7.86 ± 0.34 ^Ce^	9.70 ± 0.20 ^Bd^	11.22 ± 0.26 ^Ad^
TA (%)	Brightwell	2.04 ± 0.03 ^Cbc^	2.26 ± 0.17 ^Babc^	2.84 ± 0.03 ^Abc^	1.19 ± 0.09 ^Dd^	0.98 ± 0.02 ^Ee^	0.65 ± 0.03 ^Fd^
	Homebell	2.33 ± 0.25 ^Ba^	2.41 ± 0.07 ^Bbc^	2.75 ± 0.07 ^Ac^	1.71 ± 0.03 ^Cc^	1.23 ± 0.04 ^Dd^	0.81 ± 0.05 ^Eb^
	Emerald	1.56 ± 0.12 ^Ed^	2.53 ± 0.08 ^Bab^	3.39 ± 0.09 ^Aa^	1.95 ± 0.02 ^Cb^	1.74 ± 0.04 ^Db^	0.87 ± 0.03 ^Fa^
	Bluegold	2.32 ± 0.45 ^Ba^	2.41 ± 0.23 ^Bab^	3.34 ± 0.14 ^Aa^	2.39 ± 0.02 ^Ba^	1.88 ± 0.07 ^Ca^	0.59 ± 0.05 ^De^
	Legacy	2.17 ± 0.09 ^Cab^	2.57 ± 0.12 ^Ba^	2.89 ± 0.07 ^Ab^	1.99 ± 0.09 ^Db^	1.60 ± 0.10 ^Ec^	0.73 ± 0.02 ^Fc^
TSS/TA	Brightwell	2.53 ± 0.07 ^Dabc^	2.12 ± 0.16 ^Dcd^	2.13 ± 0.06 ^Db^	11.10 ± 0.72 ^Ca^	13.90 ± 0.30 ^Ba^	29.40 ± 2.06 ^Aa^
	Homebell	2.57 ± 0.24 ^Dabc^	2.69 ± 0.14 ^Da^	2.33 ± 0.04 ^Da^	6.50 ± 0.19 ^Cb^	9.32 ± 0.25 ^Bb^	18.60 ± 1.09 ^Ac^
	Emerald	2.72 ± 0.27 ^Dab^	1.76 ± 0.16 ^Ee^	1.58 ± 0.03 ^Ed^	4.81 ± 0.17 ^Cc^	5.43 ± 0.18 ^Bc^	12.50 ± 0.74 ^Ae^
	Bluegold	2.38 ± 0.36 ^Dc^	2.28 ± 0.22 ^Dbc^	1.82 ± 0.09 ^Dc^	4.26 ± 0.30 ^Cd^	5.63 ± 0.16 ^Bc^	20.40 ± 1.63 ^Ab^
	Legacy	2.42 ± 0.09 ^Dbc^	1.98 ± 0.22 ^DEde^	1.61 ± 0.07 ^Ed^	3.96 ± 0.28 ^Cd^	6.09 ± 0.39 ^Bc^	15.30 ± 0.65 ^Ad^

Results are expressed as mean ± SD. Different lowercase letters indicate significant differences in the same growing stage among different cultivars, while different capital letters indicate that there are significant differences among the same cultivar in the different growing stages (*p* < 0.05).

### 3.2. Changes in Soluble Sugars and Organic Acids of Five Blueberry Cultivars

#### 3.2.1. Changes in Soluble Sugar Composition

**Figure 2:**
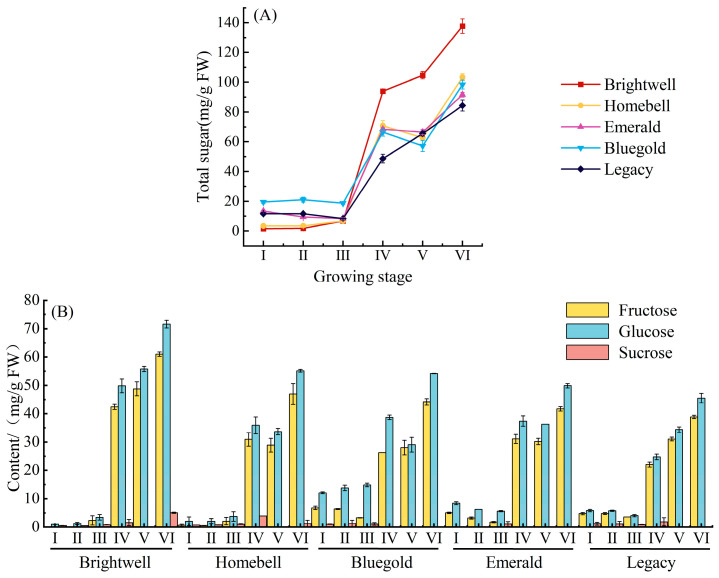
Changes in sugar content of five blueberry cultivars across six growth stages. (**A**) Changes in total sugar content. (**B**) Sugar composition at different growth stages.

The retention times, linear regression equations, linear ranges, and correlation coefficients for soluble sugars are presented in Table S2. Figure 2 shows the HPLC separation results of sugars in blueberry fruits at different growth stages. As illustrated in Figure 2A, total sugar content generally increased progressively throughout fruit maturation. At ripening stage VI, the total sugar content of the five cultivars ranked as follows: ‘Brightwell’ > ‘Homebell’ > ‘Bluegold’ > ‘Emerald’ > ‘Legacy’. As shown in Figure 2B, sucrose content in all blueberry fruits was consistently much lower than the fructose and glucose contents. The composition and changing patterns of soluble sugars in ‘Brightwell’ and ‘Homebell’ were similar: from Stages I to III, the proportion of sucrose relative to total sugar gradually decreased from 34.9% and 20.7% to 12.3% and 14.9%, respectively, while the proportions of glucose and fructose increased concurrently. At full ripeness, the rabbiteye blueberries retained a small amount of sucrose (3.68% and 1.18%), whereas sucrose was not detected in the highbush cultivars. The reducing sugar (glucose and fructose) content of each blueberry cultivar began to increase significantly from the early coloring stage (Stage IV) and reached its highest level at full maturity. Among the five cultivars, the fully ripe ‘Brightwell’ exhibited the highest fructose content, which was 29.89%, 46.29%, 38.03%, and 56.92% higher than that of the other four cultivars, respectively.

#### 3.2.2. Changes in Organic Acid Composition

**Figure 3:**
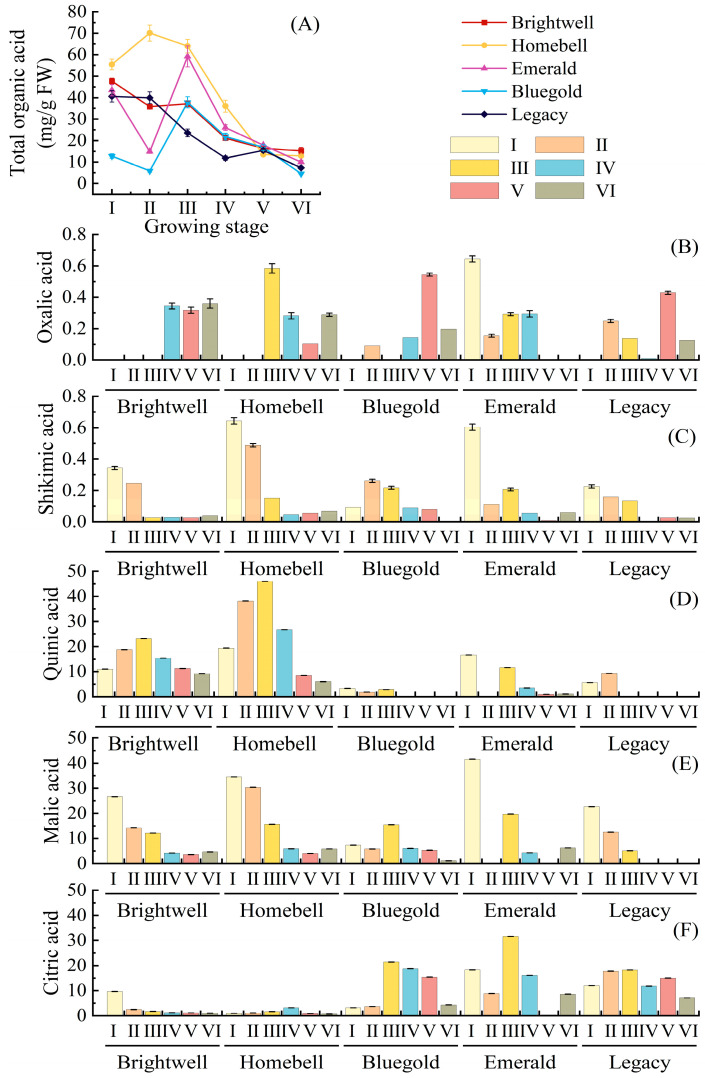
Changes in organic acid content of five blueberry cultivars across six growth stages. (**A**) Total organic acid content. (**B**–**F**) Contents of oxalic acid, shikimic acid, quinic acid, malic acid, and citric acid in the five cultivars at different growth stages.

The retention times, linear regression equations, linear ranges, and correlation coefficients for organic acids are presented in Supplementary Material Table S1. The changes in total organic acid content and the contents of five individual organic acids across different growth stages for the five cultivars are presented in Figure 3. The results showed considerable variation in organic acid composition and content among cultivars at different developmental stages. As shown in Figure 3A, total organic acid content exhibited a declining trend in all blueberry cultivars. Among fully ripe fruits, ‘Brightwell’ had the highest total organic acid content, which was 1.34, 1.52, 1.42, and 1.66 times higher than those of ‘Homebell’, ‘Bluegold’, ‘Emerald’, and ‘Legacy’, respectively. Quinic acid, malic acid, and citric acid together accounted for over 97% of the total organic acids (Figure 3D–F), whereas oxalic acid and shikimic acid were present in very low amounts (Figure 3B,C). The composition and content changes in major organic acids in rabbiteye blueberries were similar to each other but significantly different from those in highbush cultivars: in rabbiteye blueberries, malic acid and quinic acid together accounted for more than 90% of the total organic acids at all stages, with citric acid present at lower levels. As the fruit ripened, quinic acid initially increased and then decreased, while malic acid showed the opposite trend. Trace amounts of shikimic acid were detected in the early growth stages but not detected later, with small amounts of oxalic acid appearing instead. In contrast, citric acid was the predominant organic acid in mature highbush blueberries, accounting for 53.76%, 76.79%, and 97.91% of the total organic acids in ‘Emerald’, ‘Bluegold’, and ‘Legacy’, respectively, followed by quinic acid and malic acid.

### 3.3. The Correlation Analysis

**Figure 4:**
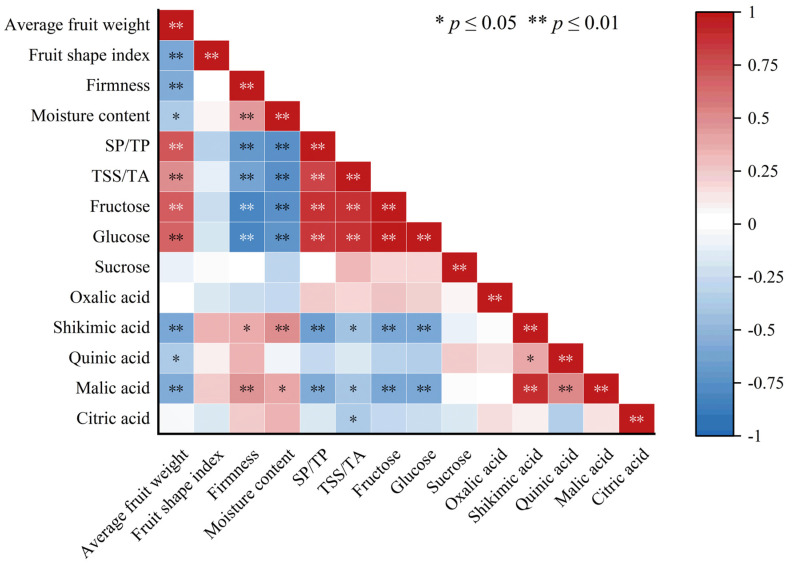
Correlation analysis of main indices of five blueberry cultivars. * indicates a significant correlation (*p* ≤ 0.05), and ** indicates a highly significant correlation (*p* ≤ 0.01).

To investigate the correlations among the main indices of the five blueberry cultivars, Pearson correlation analysis was performed. The closer the absolute value of the Pearson correlation coefficient (r) is to 1, the stronger the linear correlation between two parameters, whereas values closer to 0 indicate a weaker correlation. The results are presented in Figure 4, where red represents positive correlations and blue represents negative correlations, with darker colors indicating stronger correlations.

The correlation analysis results showed that average fruit weight was highly significantly positively correlated with SP/TP, TSS/TA, fructose, and glucose, while it was significantly negatively correlated with fruit shape index, firmness, shikimic acid, malic acid, moisture content, and quinic acid, indicating that during fruit ripening, the increase in fruit weight is consistent with the enhancement of sweetness. Fruit firmness and moisture content were significantly positively correlated with malic acid and shikimic acid, but highly significantly negatively correlated with SP/TP, TSS/TA, fructose, and glucose, suggesting that sugar accumulation is closely associated with fruit softening. With the exception of sucrose, the other two soluble sugars (fructose and glucose) were highly significantly positively correlated with TSS/TA and highly significantly negatively correlated with shikimic acid and malic acid, which may be attributable to the relatively low content of sucrose in the fruit. TSS/TA was significantly negatively correlated with shikimic acid, malic acid, and citric acid. Malic acid was positively correlated with shikimic acid and quinic acid, but showed no significant correlation with citric acid, indicating that different organic acids may follow distinct metabolic pathways during fruit ripening.

### 3.4. The Cluster Analysis

**Figure 5:**
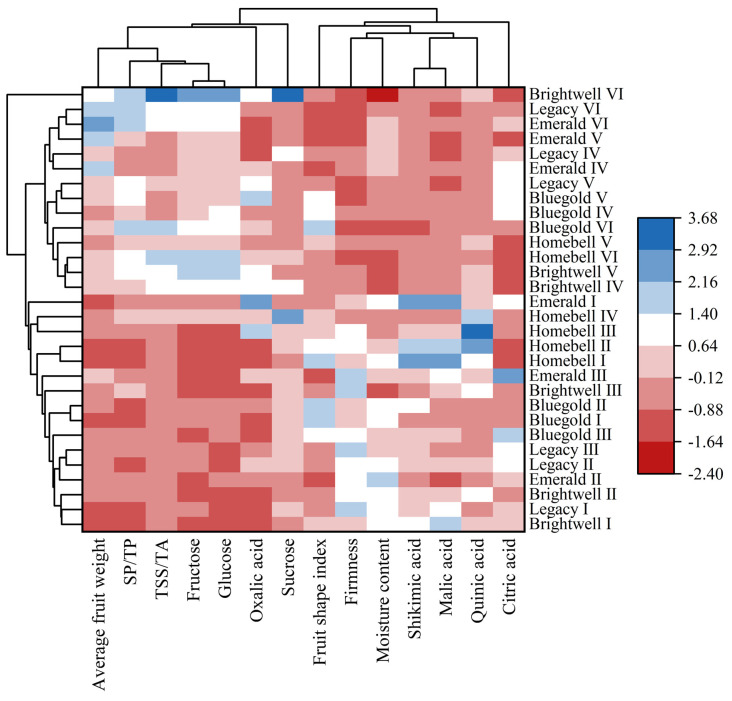
Cluster heat map of 14 quality parameters for 30 blueberry fruit samples.

Cluster analysis was performed on blueberry fruits at different growth stages and their physicochemical quality indicators, including organic acids and sugars, with the results presented as a heatmap in Figure 5. The measured indicators were classified into two main categories: Class 1 comprised average fruit weight, SP/TP, TSS/TA, and soluble sugars; Class 2 consisted of fruit shape index, firmness, moisture content, and organic acids. Based on the correlation analysis, Class 1 indicators were associated with fruit weight, texture, and taste, while Class 2 indicators were related to physical properties such as firmness and moisture content.

The five blueberry cultivars were clearly separated by growth stage into two groups: Stages I~III (from young fruit to large green fruit) and Stages IV~VI (from coloring to full ripening), indicating that fruit coloring is a critical turning point for significant changes in quality characteristics. Within Stages I~III, the highbush cultivar ‘Bluegold’ and the rabbiteye cultivar ‘Homebell’ clustered together, suggesting similar quality traits during this period. In contrast, at full ripeness, ‘Brightwell’ formed a distinct branch separate from the other cultivars, highlighting the uniqueness of its quality characteristics at the mature stage.

## 4. Discussion

Fruit maturity is a critical factor influencing the quality formation of blueberries, and different cultivars exhibit significant variation in quality change patterns during the ripening process. Together, these two factors determine the suitability of fruit for processing and consumer preference [26]. During blueberry ripening, moisture content and firmness gradually decrease, while the SP/TP ratio, which is significantly negatively correlated with both parameters, increases progressively (Figure 4), indicating fruit softening. This is associated with the gradual breakdown of protopectin into soluble pectin by pectinases [27]. As in most fruits, soluble sugars in blueberries are primarily composed of fructose and glucose. Once blueberries enter the coloring stage (Stage IV), starch begins to hydrolyze into soluble sugars, leading to a marked increase in total soluble sugar content (Figure 2A), in agreement with earlier studies [28], which is mainly driven by the accumulation of fructose and glucose, both of which share highly similar accumulation patterns (Figure 4). Sucrose content remains very low and further decreases or disappears entirely (Figure 2B). This is attributed to enhanced invertase activity during late-stage development, which partially or completely converts sucrose into reducing sugars, primarily fructose and glucose [29,30,31], consistent with their coordinated accumulation profiles. Moreover, previous studies have indicated that the downregulation of VcSWEET6a expression during blueberry ripening also contributes to the increase in reducing sugar content [32]. This finding is consistent with the cluster analysis, where the changing trends of SP/TP, TSS/TA, and soluble sugars were closely similar (Figure 5). During fruit development, total organic acid content peaks at the large green fruit stage (Stage III) and subsequently declines (Figure 3A), which is in agreement with previous findings [15]. Among organic acids, citric acid, malic acid and qunic acid are the predominant components and serve as important substrates for energy production via the tricarboxylic acid (TCA) cycle [33]. In the early growth stages, blueberries continuously accumulate organic acids and starch. As development progresses into mid-to-late stages, the phosphoenolpyruvate carboxylase (PEPC) gene becomes significantly expressed [34,35], leading to the consumption of malic acid and citric acid as respiratory substrates. Consequently, their contents decline markedly (Figure 3E,F). Among them, the decline in malic acid was negatively correlated with the increase in single fruit weight and moisture content (Figure 4). In the cluster analysis, these parameters were grouped together, confirming the correlation among their changes (Figure 5). Quinic acid, a precursor for the synthesis of phenolic compounds such as anthocyanins [36,37], is also utilized after the onset of fruit coloring. The changing patterns of these three major organic acids in blueberries are similar (Figure 4). Collectively, these observations indicate that organic acids not only influence fruit taste but also play an essential role in fruit growth and development [38]. Thus, during blueberry ripening, organic acids and soluble sugars assume distinct metabolic roles: most organic acids are consumed as respiratory substrates, whereas soluble sugars are primarily derived from the hydrolysis of polysaccharides and disaccharides. As a result, fruit taste becomes progressively sweeter with advancing maturity.

This study analyzed the quality variation patterns of five blueberry cultivars during growth and development, revealing that their quality characteristics clustered according to cultivated type (rabbiteye vs. highbush) (Figure 5). In terms of physicochemical properties, rabbiteye blueberries exhibited significantly lower moisture content and SP/TP ratio than highbush blueberries, and with the exception of ‘Legacy’, they also showed higher firmness and more seeds (Figure 1). This may be attributed to the fact that rabbiteye blueberries, as a late-ripening cultivated type, undergo a longer fruit development period and are more exposed to biotic and abiotic stresses, thereby retaining wilder traits [39]. In addition, the results showed that quinic acid content was higher in rabbiteye blueberries than in highbush blueberries (Figure 3), which is consistent with previous findings [40]. It has been reported that interspecific differences in quinic acid content are primarily governed by genetic background, with wild diploid blueberries containing significantly higher quinic acid levels than cultivated cultivars [41]. As late-ripening cultivated types, rabbiteye blueberries undergo a longer fruit development period and are more exposed to biotic and abiotic stresses. Therefore, it is hypothesized that rabbiteye blueberries may preferentially synthesize quinic acid to enhance stress tolerance, which could explain the higher quinic acid content observed in this type compared to highbush blueberries in this study [36,42,43]. In addition, our results for ‘Bluegold’ differ from those of Zhang et al. [10], who reported citric and quinic acids as the major organic acids, whereas we found malic acid to be the second most abundant, which is likely due to regional or environmental factors.

The composition and content of soluble sugars and organic acids are important indicators for evaluating fruit quality and taste [44]. The sweetness of fruit is determined by its soluble sugar composition. Fructose is sweeter than sucrose and glucose, with relative sweetness values of 1.75, 1.0, and 0.7, respectively; thus, a higher fructose content contributes to a sweeter taste [8]. Similarly, organic acid composition influences fruit acidity. Among the five organic acids analyzed, malic acid exhibits the highest sourness intensity, followed by citric acid [12]. Among the five cultivars, ‘Brightwell’ and ‘Homebell’ exhibited high TSS/TA values and fructose content at ripening stage VI, suggesting a sweeter taste for fresh consumption. Additionally, both cultivars showed higher firmness and lower moisture content compared to highbush blueberries, making them more suitable for storage and transportation. In contrast, the three highbush cultivars maintained higher citric acid levels, and since citric acid was significantly negatively correlated with TSS/TA, this implies a more acidic taste, which may appeal to consumers preferring a refreshing taste. Highbush blueberries exhibited lower firmness and softer texture, making them less suitable for long-term storage but ideal for fresh consumption or processing into juice and jam, consistent with previous findings [11]. Furthermore, ‘Bluegold’ displayed a higher fruit shape index and rounder fruit shape, indicating superior appearance quality, while ‘Legacy’ and ‘Emerald’ had larger fruit weight, enhancing their commercial value. Overall, rabbiteye blueberries are characterized by high sugar content and firmness, making them suitable for long-term storage and processing, whereas highbush blueberries offer a balanced sweet–sour taste and juicy texture, making them preferable for fresh consumption and juice processing. These cultivar-specific differences provide a theoretical basis for breeding programs aimed at meeting diverse consumer preferences and processing needs.

## 5. Conclusions

This study revealed that the sugar–acid composition of blueberry fruits is dependent on cultivars, with glucose and fructose as the main soluble sugars, malic and citric acids as the predominant organic acids, and quinic acid uniquely accumulating in rabbiteye blueberries, likely associated with their late-ripening genotype. In contrast, the contents of quinic acid, malic acid, and citric acid decreased during ripening. Among them, malic acid showed a significant positive correlation with fruit firmness and moisture content. Distinct differences in the TSS/TA values and sugar–acid composition were observed between rabbiteye and highbush blueberries. ‘Brightwell’ and ‘Homebell’ exhibited higher TSS/TA and firmness, resulting in a sweeter taste and better storage tolerance. ‘Bluegold’, ‘Emerald’, and ‘Legacy’ showed softer texture and higher moisture content, together with a balanced TSS/TA ratio and sugar–acid composition, suggesting their potential suitability for fresh consumption or processing into juice and jam. This study not only elucidates the changes in physicochemical properties and sugar–acid components in blueberries at different growth stages, but also provides a reference for consumer selection and for growers to determine the optimal harvest time. Our future research should validate these findings under different environmental conditions and integrate antioxidant analysis, in addition to combining sensory evaluation with transcriptomic approaches to further elucidate the molecular mechanisms underlying cultivar-specific sugar–acid dynamics.

## Data Availability

The original contributions presented in the study are included in the article/Supplementary Materials, further inquiries can be directed to the corresponding author.

## Supplementary Materials

The following supporting information can be downloaded at https://www.mdpi.com/article/10.3390/foods15122157/s1, Note S1: Determination of the moisture content of blueberry fruits; Note S2: Determination of the pectin content of blueberry fruits; Table S1: Range of days after full bloom (DAFB) for each cultivar at six developmental stages; Table S2: The retention time, linear equation, linearity range, correlation coefficient of organic acids and sugars.

## Abbreviations

The following abbreviations are used in this manuscript:

SP	Soluble Pectin
TP	Total Pectin
TSS	Total Soluble Solids
TA	Titratable Acid
HPLC	High Performance Liquid Chromatography
