# Supplementary material for: Changes in Fruit Characteristics of Various Blueberry Cultivars During Ripening Stages
Source: Foods. 2026 Jun 15;15(12):2157. doi: 10.3390/foods15122157 (PMC13298151; doi:10.3390/foods15122157)
Supplement: Supplementary file 1 [file foods-15-02157-s001.zip › foods-4323564-supplementary.pdf]

Supplementary material

Changes in fruit characteristics of various blueberry cultivars during ripening stages

The file includes:

A. Supplementary notes

B. Supplementary tables

A. Supplementary notes

Notes S1: Determination of the moisture content of blueberry fruits.

Take a clean glass weighing bottle, place it in a drying oven at 101 °C to 105 °C with the lid tilted against the bottle rim, and heat for 1 h. After removal, cover the bottle and allow it to cool in a desiccator for 0.5 h before weighing. Repeat the drying process until the difference between two consecutive weighing is no greater than 2 mg, which is considered constant weight. Rapidly grind 10 blueberries of the same variety and growth stage to a particle size less than 2 mm. Accurately weigh 2 g blueberry sample with 0.0001 g precision , place it in the weighing bottle, cover, and record the precise mass. Then place the bottle in the drying oven at 101 °C to 105 °C with the lid tilted, and dry for 4 h. After drying, cover the bottle, remove it, and cool in a desiccator for 0.5 h before weighing. Subsequently, return the bottle to the drying oven and dry for approximately 1 h. Remove, cool in the desiccator for 0.5 h, and weigh again. Repeat the procedure until the mass difference between two successive weighing is within 2 mg, indicating constant weight.

The water content in the blueberry sample is calculated according to the following equation:

$$\text{Water content}(\%)=\frac{m_1-m_2}{m_1-m_3}\times 100$$

Where m_1, m_2 are the initial and final weight (g) of weighing bottle and blueberry sample,respectively. m_3 is the weighing bottle weight (g). The calculation results are rounded to two significant digits. Each group of blueberry samples was re-tested three times.

Note S2: Determination of the pectin content of blueberry fruits

The soluble pectin and total pectin contents in blueberry fruits were determined by the cyclazole colorimetric method. After the fresh blueberry fruits were washed successively with tap

water and deionized water, the surface moisture was gently wiped off with clean gauze. Several blueberry fruits of each variety and each growth period were randomly selected and crushed and mixed evenly. They were then placed in a tissue homogenizer to make a homogenate, which was frozen at -20℃ for storage.

Weigh 2.5 g (accurate to 0.001 g) of the blueberry mixture into a 50 mL graduated centrifuge tube. A small amount of filter paper and 35 mL of preheated to 75℃ anhydrous ethanol were added, and the mixture was shaken and heated in an 85℃ water bath for 10 minutes. After cooling, anhydrous ethanol was added to make the total volume close to 50 mL. The mixture was centrifuged at 4000 r/min for 15 minutes, and the supernatant was discarded. The precipitate was washed with 67% ethanol solution in an 85℃ water bath, centrifuged, and the supernatant was discarded. This process was repeated until there was no sugar reaction in the supernatant. The resulting precipitate was the crude pectin extract. A blank control of the reagents was also set up.

The total pectin extraction involves washing the above precipitate with 0.5 pH sulfuric acid solution into a beaker, mixing well, heating in a water bath at 85°C for 60 minutes, cooling, transferring to a 100 mL volumetric flask, adjusting the pH to 0.5 with sulfuric acid solution, diluting, filtering, and using the filtrate for total pectin content determination. The extraction of soluble pectin involves washing the above precipitate with an appropriate amount of hot water into a beaker, shaking at room temperature for 30 minutes, transferring to a 100 mL volumetric flask, diluting with water, filtering, and using the filtrate for soluble pectin content determination.

Accurately transfer 1.0 mL of each of 0.0, 20.0, 40.0, 60.0, 80.0, and 100.0 mg/L galacturonic acid standard solution into a 25 mL test tube, add 0.25 mL of 1 g/L coumarin ethanol solution, shake well, quickly add 5.0 mL concentrated sulfuric acid, and immediately shake and mix. Place the test tube in an 85°C water bath shaker for reaction for 20 minutes, remove and quickly cool in an ice bath within 1.5 hours, then measure the absorbance at 525 nm wavelength, and plot a standard curve with galacturonic acid concentration as the abscissa and absorbance value as the ordinate. Transfer 1.0 mL of the test filtrate into a 25 mL test tube, perform the reaction according to the above method, and measure the absorbance at 525 nm. The galacturonic acid concentration in filtrate is calculated according to the standard curve,and the pectin content in the blueberry sample is calculated according to the following equation:

$$\text{pectin content(mg GAE/100 g FW)} = \frac{\rho \times V}{m \times 1000}$$

Where ρ, V and m are the galacturonic acid concentration in filtrate, volume for pectin precipitation volume after dilution and blueberry sample weight, respectively.

B. Supplementary tables

Table S1

Range of days after full bloom (DAFB) for each cultivar at six developmental stages.

Ripening Stage	Brightwell	Homebell	Bluegold	Emerald	Legacy
Stage I	25~27	25~27	25	25	25
Stage II	35~37	35~37	35	35	35
Stage III	45~47	45~47	45	45	45
Stage IV	55~58	55~58	55	55	55
Stage V	60~63	60~63	60	60	60
Stage VI	65~68	65~68	65	65	65

Table S2

The retention time, linear equation, linearity range, correlation coefficient of organic acids and sugars.

Components	Retention time(min)	Linear equation	Linearity range (μg/mL)	Correlation coefficient (R^2)
Fructose	7.129	Y=29.941x-3.151	20~250	0.9997
Glucose	7.945	Y=19.572x-1.807	20~250	0.9992
Sucrose	9.968	Y=35.74x-3.1153	20~250	0.9997
Oxalic acid	3.796	Y=7099.3x-495.49	1~20	0.9993
Quinic acid	5.125	Y=219.1x+3301.1	50~1000	0.9994
Shikimic acid	5.926	Y=37195x-1230.7	1~20	0.9999
Malic acid	6.644	Y=496.81x-2814.9	50~1000	0.9999
Citric acid	14.739	Y=841.08-3959	50~1000	0.9999
